# Risk factors associated with cytomegalovirus infection in patients with drug-induced hypersensitivity syndrome/drug rash with eosinophilia and systemic symptoms (DIHS/DRESS)

**DOI:** 10.1016/j.jdin.2025.01.009

**Published:** 2025-01-31

**Authors:** Fumi Miyagawa, Yoshiko Mizukawa, Yasuaki Hayashino, Yuki Nakamura-Nishimura, Hideo Asada

**Affiliations:** a Department of Dermatology, Nara Medical University School of Medicine, Nara, Japan; b Department of Dermatology, Kyorin University School of Medicine, Tokyo, Japan; c Department of Endocrinology, Tenri Hospital, Nara, Japan

**Keywords:** CD57, CD8 T-cell count, CMV antigenemia, CMV disease, creatinine, CX3CR1, drug eruption, drug-induced hypersensitivity syndrome (DIHS)/drug rash with eosinophilia and systemic symptoms (DRESS), epidemiology, neutrophil-to-lymphocyte ratio (NLR), systemic corticosteroids

*To the Editor:* Drug-induced hypersensitivity syndrome (DIHS)/drug rash with eosinophilia and systemic symptoms (DRESS) is a severe systemic cutaneous adverse drug eruption, in which cytomegalovirus (CMV) disease is a major prognostic indicator.^1^^,^^2^ However, the factors influencing the progression to CMV disease are poorly understood, and several attempts have been made to predict the development of CMV disease in DIHS/DRESS, including the establishment of a scoring system, the DIHS/DRESS severity score.^1^ However, this system does not include hematologic parameters, such as lymphocyte subset counts. Since immunosurveillance, which is predominantly mediated by CD8 T cells, is essential for preventing CMV replication,^3^ we sought to identify laboratory predictors of CMV disease including lymphocyte subsets in DIHS/DRESS.

Blood samples were collected at the initial presentation from 84 patients with drug eruptions (DIHS/DRESS: 35, Stevens-Johnson syndrome/toxic epidermal necrolysis: 8, maculopapular exanthema: 41) and analyzed as described in the Supplementary Information, available via Mendeley at https://data.mendeley.com/datasets/2jdy2wr2bz/1.

We first evaluated all 84 drug eruption patients. Of these, 11 exhibited CMV antigenemia (DIHS/DRESS: 8, Stevens-Johnson syndrome/toxic epidermal necrolysis: 1, maculopapular exanthema: 2), and 6 DIHS/DRESS patients developed CMV disease. The patients with CMV disease/antigenemia were older (72.3 vs 62.8 years) and delayed their initial visits. They received higher total corticosteroid doses in the 8 weeks after the initial presentation (1265 vs 450 mg) and had higher neutrophil-to-lymphocyte ratios (6.1 vs 4.1) and creatinine (Cr) levels (1.23 vs 0.77 mg/dL) (Fig 1, Supplementary Table I, available via Mendeley at https://data.mendeley.com/datasets/2jdy2wr2bz/1).

**Fig 1:**
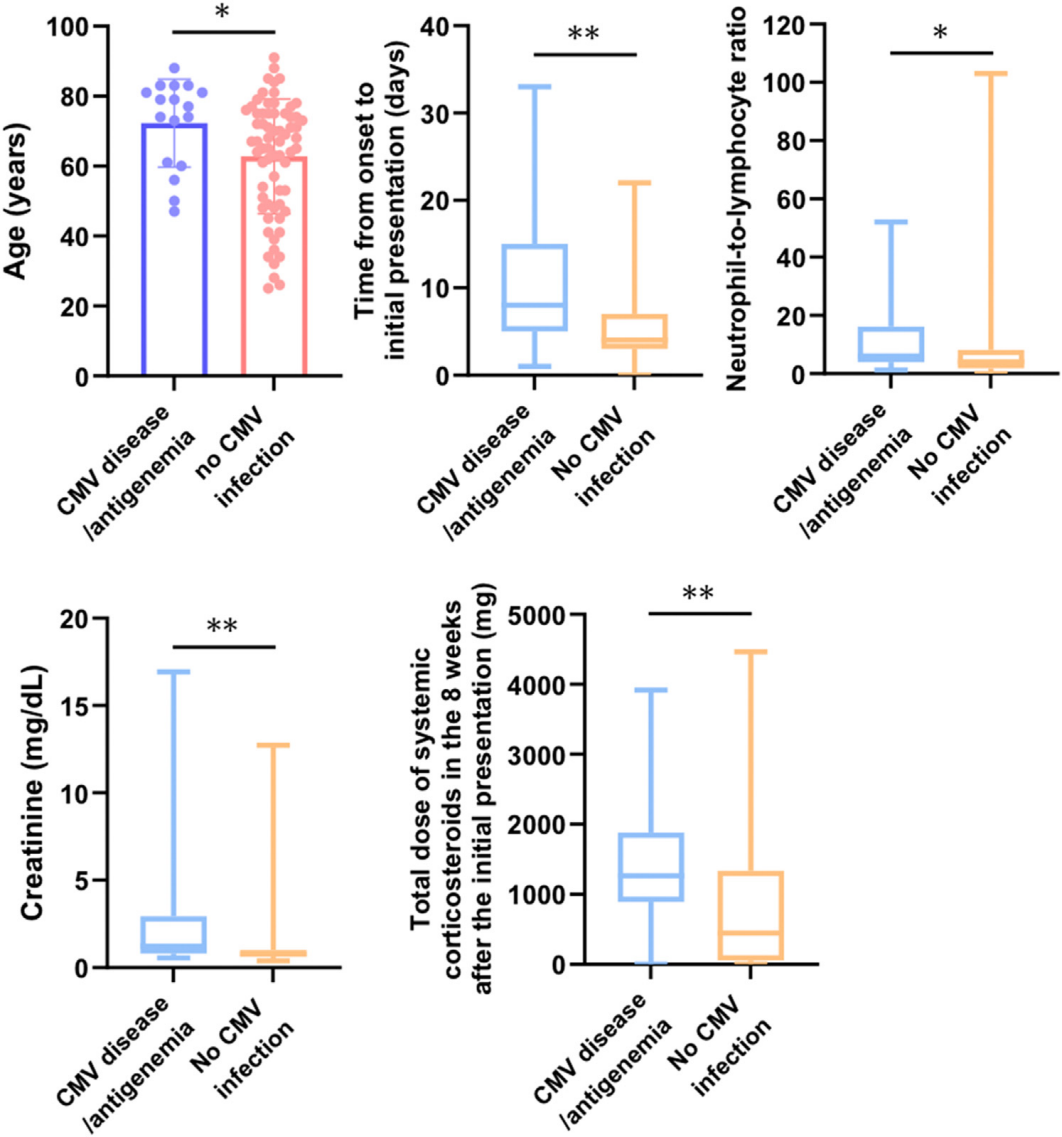
Clinical characteristics and laboratory parameters that exhibited significant differences between the patients with CMV disease/antigenemia (*n* = 17) and those without CMV infections (*n* = 67) among 84 patients with drug eruptions. The first bar graph demonstrates the mean ages of the 2 groups, and the error bars represent SD. In the other 4 box plots, the lines in the middle of the boxes indicate the median value. The ends of the boxes demarcate the 25th to 75th percentiles, and the whiskers extend to the smallest and largest values. ∗*P* < .05, ∗∗*P* < .01. *CMV*, Cytomegalovirus.

We next extracted DIHS/DRESS patients because the incidence of CMV disease is markedly higher in DIHS/DRESS patients, and those who did not receive corticosteroids also developed CMV disease (Supplementary Table II, available via Mendeley at https://data.mendeley.com/datasets/2jdy2wr2bz/1), suggesting that DIHS/DRESS itself causes immunosuppression and DIHS/DRESS patients may have different risk factors than the whole study population. Of the 35 DIHS/DRESS patients, 6 developed CMV disease and 8 exhibited CMV antigenemia. Similar to the whole study population, the 14 patients with CMV disease/antigenemia were older (71.8 vs 58.6 years) and had higher Cr levels (1.27 vs 0.74 mg/dL). Importantly, they exhibited significantly lower CD8 T-cell counts (157.5 vs 522/μL), higher CD4/CD8 ratios (2.99 vs 1.37), lower CX3CR1^+^CD57^+^CD8 T-cell counts (17.1 vs 66.8/μL), and higher DIHS/DRESS severity scores than the patients without CMV infections (Fig 2, Supplementary Table II, available via Mendeley at https://data.mendeley.com/datasets/2jdy2wr2bz/1). This suggests that DIHS/DRESS patients with low CD8 and CX3CR1^+^CD57^+^CD8 T-cell counts are more likely to develop CMV infections. The number of terminally differentiated senescent CD57^+^CD8 T cells, which exhibit upregulated CX3CR1 expression, a marker of memory CD8 T cells with cytotoxic effector function, is increased in CMV-infected individuals.^4^^,^^5^

**Fig 2:**
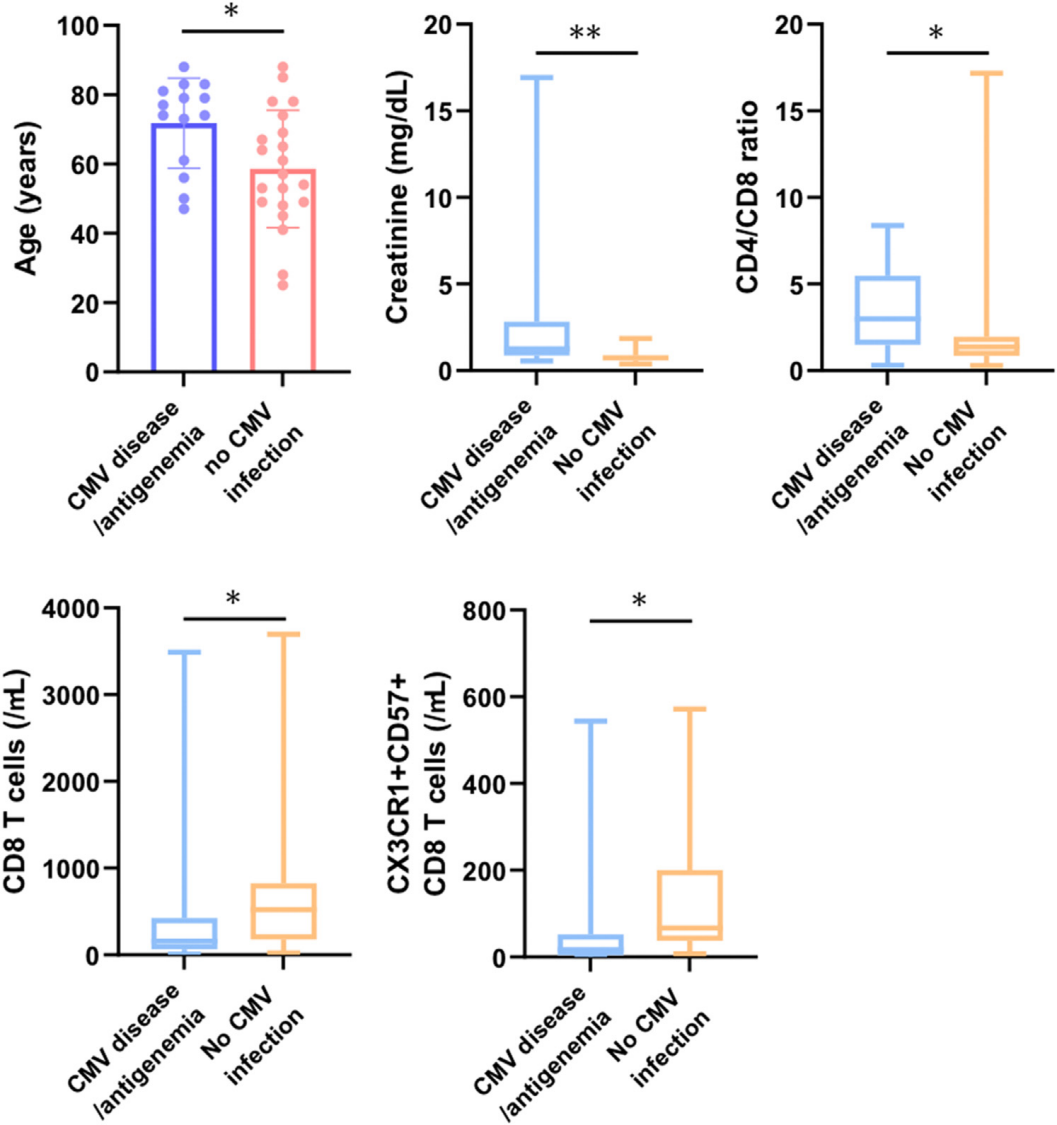
Clinical characteristics and laboratory parameters that exhibited significant differences between the patients with CMV disease/antigenemia (*n* = 14) and those without CMV infections (*n* = 21) among 35 patients with DIHS/DRESS. The first bar graph demonstrates the mean ages of the 2 groups, and the error bars represent SD. In the other 4 box plots, the lines in the middle of the boxes indicate the median value. The ends of the boxes demarcate the 25th to 75th percentiles, and the whiskers extend to the smallest and largest values. ∗*P* < .05, ∗∗*P* < .01. *CMV*, Cytomegalovirus; *DIHS/DRESS*, drug-induced hypersensitivity syndrome/drug rash with eosinophilia and systemic symptoms.

In addition to the well-known factors identified for drug eruption patients, such as the total corticosteroid dose, a low CD8 T-cell count at the initial presentation was identified as an independent predictor of CMV disease in DIHS/DRESS. CMV seropositivity and CD8 T-cell count monitoring at the initial presentation are additional tools for assessing the risk of CMV disease developing in DIHS/DRESS.

Our study’s main limitation is that it merely identified factors associated with more severe disease without changing management practices because all patients need to be monitored for CMV reactivation anyway.

## Conflicts of interest

None disclosed.
